# The transcription factor PdePHR1 increases phosphorus accumulation in poplar shoot and promotes root growth

**DOI:** 10.1186/s12870-026-09584-6

**Published:** 2026-07-24

**Authors:** Qiang Wang, Lingfeng Chen, Dandan Jia, Fei Wang, Tao Pu, Wenfeng Li

**Affiliations:** Co-Innovation Center for Sustainable Forestry in Southern China, College of Life Sciences, Nanjing Forestry University, Nanjing, 210037 China

**Keywords:** Populus, Phosphorus, PHR1, Root, Pi deficiency tolerance, Growth

## Abstract

**Background:**

Trees are major primary producers and long-term carbon sink by yielding a substantial amount of woody biomass, which relies on sufficient acquisition of mineral nutrient from soil by roots. Phosphate (Pi) is an essential, but often limited in soils, macronutrient. To sustain growth during Pi starvation, plants trigger an array of adaptive responses that are mainly controlled by the transcription factor of the PHOSPHATE RESPONSE 1 (PHR1) family. However, how the PHR1 functional homolog might influence the performance of forest trees remains unknown.

**Results:**

In this work, we identified PdePHR1from *Populus deltoides* x *P. euramericana* as a MYB-CC type transcription factor. Furthermore, overexpression of PdePHR1 in *P. davidiana* x *P.bolleana* triggers the expression of the Pi starvation response genes in replete Pi conditions, including genes mediating Pi uptake, remobilization, and signaling. Moreover, plants with overexpression of PdePHR1 exhibit enhanced Pi acquisition and translocation, leading to higher phosphorus accumulation in shoots under optimal conditions. Additionally, PdePHR1 overexpressing lines display substantially longer adventitious roots and more biomass accumulation independent of external Pi availability. Furthermore, overexpression of PdePHR1 sufficiently improves poplar performance under both Pi-replete and long-term depleted conditions.

**Conclusions:**

Our data support that PdePHR1 acts as a central positive regulator of Pi starvation response and root growth. PdePHR1 overexpression is sufficient to enable not only increased phosphorus accumulation in shoot but also greatly enhanced root growth and overall woody biomass accumulation regardless of Pi availability. These findings provide a potential tool for increasing tree productivity and sustainable carbon sequestration under various environmental conditions.

**Supplementary Information:**

The online version contains supplementary material available at https://doi.org/10.1186/s12870-026-09584-6.

## Background

Forest trees are major driver of carbon stock in woody biomass, which contribute strongly to mitigate climate change [16]. However, limited nutrient availability in soils puts constraint on tree growth [27]. Giant trees require a significant amount of phosphorus (P) in the form of orthophosphate (Pi, H_2_PO_4_^−^/HPO_4_^2−^) to build nucleic acids, lipid membranes, and ATP in cells and to participate in carbon metabolism and protein regulation [8]. Thus, sufficient acquisition of Pi from soil is essential for plant growth and a key determinant of yield [5]. However, Pi is often immobilized by inorganic cations, organic matters and soil particles, which leads to low Pi availability [10, 28].

To cope with low Pi deficiency, plants undergo dramatic adaptive changes at morphological, physiological and molecular levels, which are collectively called Pi starvation response (PSR) [4]. Pi limitation causes a significant reduction in shoot growth and the extension of lateral roots to expand soil exploration capacity [18].As a result, the root-to-shoot ratio is increased during Pi depletion [7]. In order to enhance Pi absorption and utilization, plants produce more organic acids and acid phosphatase (APase) to release Pi from immobilized forms. Roots take up external Pi through PHOSPHATE TRANSPORTER1 (PHT1) family proteins and translocate to shoot by efflux transporter PHOSPHATE 1 (PHO1) [9, 19, 23]. The protein levels of PHT1 and PHO1 are modulated by an ubiquitin E2 conjugase PHOSPHATE 2 (PHO2) [1].

In general, the extensive transcriptional reprogramming that underlies PSR is largely directed by a master transcription factor PHOSPHATE RESPONSE 1 (PHR1) AtPHR1 in Arabidopsis (*Arabidopsis thaliana*) or its functional ortholog OsPHR2 in rice (*Oryza sativa*) (called PHR hereafter) [2, 26, 38]. PHR proteins share an MYB domain that confers target gene binding and a coiled-coil (CC) that mediates PHR dimerization. Interestingly, neither the transcription nor the protein level of PHR is changed in response to Pi availability [12]. Instead, PHR activity is modulated by cellular P status through interaction with the SPX domain-containing proteins (SPXs). Essentially, when P is sufficient, SPX physically interacts with PHR, which in turn represses its transcriptional activity. Conversely, low P levels prevent the formation of SPX-PHR complex and concomitantly lead to the expression of PSR genes [24, 32, 34].

As a core regulator, PHR transcription factors govern the expression of different types of PSR genes by binding to their PHR1-binding sequence (P1BS) as a dimer. For instance, receptors *SPX*s, long noncoding RNA *IPS1*, Pi transporters *PHT1* and purple acid phosphatases (*PAP*s) genes are well documented PHR target genes [2, 33]. Moreover, PHR might act as critical factor for the Pi starvation-mediated root system growth and development, including primary root growth, lateral root development and root hair formation [6, 33]. Together, these studies, which are predominately in herbaceous plants, highlight the essential role of PHR in translating information on Pi levels into actions to survive Pi starvation. However, the functional homolog of the master regulator PHR in forest trees remains to be elucidated.

Trees are facing the challenge of Pi scarcity worldwide. Growth rates of trees increase significantly with increasing external Pi supply [3, 30]. Therefore, promoting absorption of Pi by roots may boost sustainable tree production and ultimately help to ameliorate climate change. Poplars are fast-growing woody plants, which have great potential applications in CO_2_ mitigation [20]. Here, we identified and characterized a key PSR regulator *PdePHR1* in poplar, whose overexpression enables not only increased phosphorus accumulation in shoot but also greatly enhanced root growth and overall productivity.

## Methods

### Plant materials and growth conditions

*Populus deltoides* Bartr. cv. I-69 × *P. euramericana* cv. I-45 clone Nanlin 895 (NL895) and *P. davidiana* × *P.bolleana* were generously provided by Dr. Qiang Zhuge's research group at the State KeyLaboratory of Tree Genetics and Breeding, Nanjing Forestry University.

used in this study. Seedlings were cultivated in MS solid medium (1.25 mM Pi) at 25℃ under a light/dark cycle (16 h light/8 h dark, 4,500 lx). For NL895 phenotyping, the 15d-old young seedlings were transferred into hydroponic Hoagland medium containing 1000, 10, 2 and 0 µM Pi, respectively. The plants grew for 30 days with changing fresh medium every 3 days. For the temporal-spatial expression patterns of gene under -Pi conditions, rooted NL895 cuttings were cultured in -Pi MS medium for nine days. Different tissues including root, stem, bottom leaf and top leaf were collected at day0, 1, 3, 5 and 7 for reverse transcription quantitative PCR (RT-qPCR) analysis. To investigate the phenotype of *P. davidiana* × *P.bolleana* under normal and phosphorus starvation conditions in tubes, 3-week-old rooted cuttings with similar height were transplanted into sterilized tubes with liquid Hoagland medium with (1.25 mM Pi) or without Pi for 2 weeks. For the plants grown in green house, we first cultured wild-type and PdePHR1-overexpressing cuttings in MS medium for 25 days, and then transferred the similar seedlings into pot (vermiculite: perlite = 3:1). The seedlings were supplied with liquid 1/2 MS medium with or without Pi, respectively, watering every 3 days for 60 days.

### Identification and cloning of *PdePHR1* gene

We searched in the genome of *Populus trichocarpa* (https://phytozome-next.jgi.doe.gov) using the amino acid sequence of AtPHR1 by BLASTP program (https://www.ncbi.nlm.nih.gov/BLAST/). The DNA sequence of the best hit *Potri.002G257800.1* (*PtPHR1*) was retrieved from the database and used for primer design (Table S1). Total RNA was isolated from the leaves of NL895 using Total RNA Isolation Kit (Vazyme, Nanjing,China) and cDNA was synthesized using the HiScript III All-in-one RT SuperMix Perfect (Vazyme, Nanjing, China). Full-length coding sequence of *PdePHR1* was amplified using the following condition: 95 °C for 5 min followed by 35 cycles of 95 °C for 30 s, 59 °C for 30 s, 72 °C for 1 min 30 s, and a final extension at 72 °C for 10 min. The PCR products were purified with SanPrep Column DNA Gel Extraction Kit (Sangon, Shanghai, China) and cloned into a pEASY®-Blunt Zero plasmid. The gene was verified by sequencing and subcloned into *pCAMBIA1300-GFP* Vector with the One Step Cloning Kit (Vazyme, Nanjing,China) to construct *p35S::PdePHR1* binary vector.

### Phylogenetic analysis

The protein sequences of PHR1 homologs from different plant species, including *A. thaliana* (At), *prunus avium* (Pa), *Prunus mume* (Pm), *Prunus persica* (Pp), *Pyrus x bretschneideri* (Pb), *Malus domestica* (Md), *Medicago truncatula* (Mt), *Brassica napus* (Bn), *Oryza sativa* (Os), *Zea mays* (Zm), *Phaseolus vulgaris* (Pv), *Glycine max* (Gm), and *Chlamydomonas reinhardtii* (Cr) were retrieved from the NCBI database. MEGA 11 (https://www.megasoftware.net/) software was used to construct a phylogenetic tree based on the alignments using the neighbor-joining tree (NJT) method, replicated 1000 times.

### Subcellular localization

The coding sequence of PdePHR1 without the stop codon was cloned into the *pCAMBIA1300-GFP* vector controlled by the cauliflower mosaic virus (CaMV) 35S promoter (*35S::GFP*) to generate a *35S::PdePHR1-GFP* expression cassette and transformed into Arabidopsis. The transgenic seedlings were used for protoplast isolation as previously described [37]. GFP fluorescence was observed with Zeiss LSM710 confocal microscopy. The excitation wavelength used for GFP was 488 nm and emission filters were 500–530 nm for GFP, 680–700 nm for chlorophyll.

### Transactivation activity assay

Transactivation activity assay of PdePHR1 was performed by using AH109-GAL4 Two-Hybrid System (Coolaber, Beijing, China). The coding region of *PdePHR1* was inserted into the *pGBKT7* vector. Primers were listed in Table S1. The *pGBKT7-PdePHR1* and the negative control *pGBKT7* constructs were transformed into yeast strain AH109, respectively. The transformed strains were grown on the medium of SD/− Trp and selected on the medium of SD/-Trp-His-Ade.

### Poplar transformation

The binary construct *p35S::PdePHR1* was introduced into *Agrobacterium* strain EHA105. The Agrobacterium-mediated transformation of *P. davidiana* × *P.bolleana* was carried out using a leaf disc method following the previous report [35].

### Selection of positive transgenic plants using PCR

The positive transgenic poplar plants were screened using hygromycin and confirmed the presence of *PdePHR1* fragment insertion by PCR with vector-specific primers PdePHR1F/GFP-89R (Table S1) using genomic DNA as template. DNA extraction was conducted using a Pure Plant Genomic DNA Kit (Tsingke Biotech,Beijing,China). The gene expression level was determined by real-time quantitative reverse transcription PCR (RT–qPCR).

### RNA extraction and RT–qPCR analysis

The RNA was extracted using Total RNA Isolation Kit (Vazyme, Nanjing, China), the complementary DNA was synthesized using HiScript III All-in-one RT SuperMix Perfect (Vazyme, Nanjing, China), and PCR reactions were carried out using the CFX96 Real-Time System (Bio-Rad) with ChamQ SYBR qPCR Master Mix (Vazyme, Nanjing, China). The gene expression levels were normalized to reference EIF4B. The primers used for qPCR are listed in Table S3.

### Measurement of P content

To measure P content, 3-week-old rooted cuttings with a height of ~ 5 cm were transplanted into Pi-sufficient (1.25 mM Pi) or Pi-deficient (0 μM Pi) medium for six weeks. Plant tissues were dried at 65⁰C for three days and then weighted prior to digest with nitric acid. The total P concentration was measured using the ICP-MS method and normalized by dry weight. The measurement of total P concentration was performed by Jiangsu Science Testing Co.,LTD.

### Statistical analysis

The significance of differences between data sets was evaluated using one-way ANOVA and two-way ANOVA (*p* < 0.05, Fisher's LSD) with prism software (Graph Pad, USA). At least three independent biological replicates were included in each analysis.

## Results

### Pi deficiency severely restricts poplar growth

**Fig. 1:**
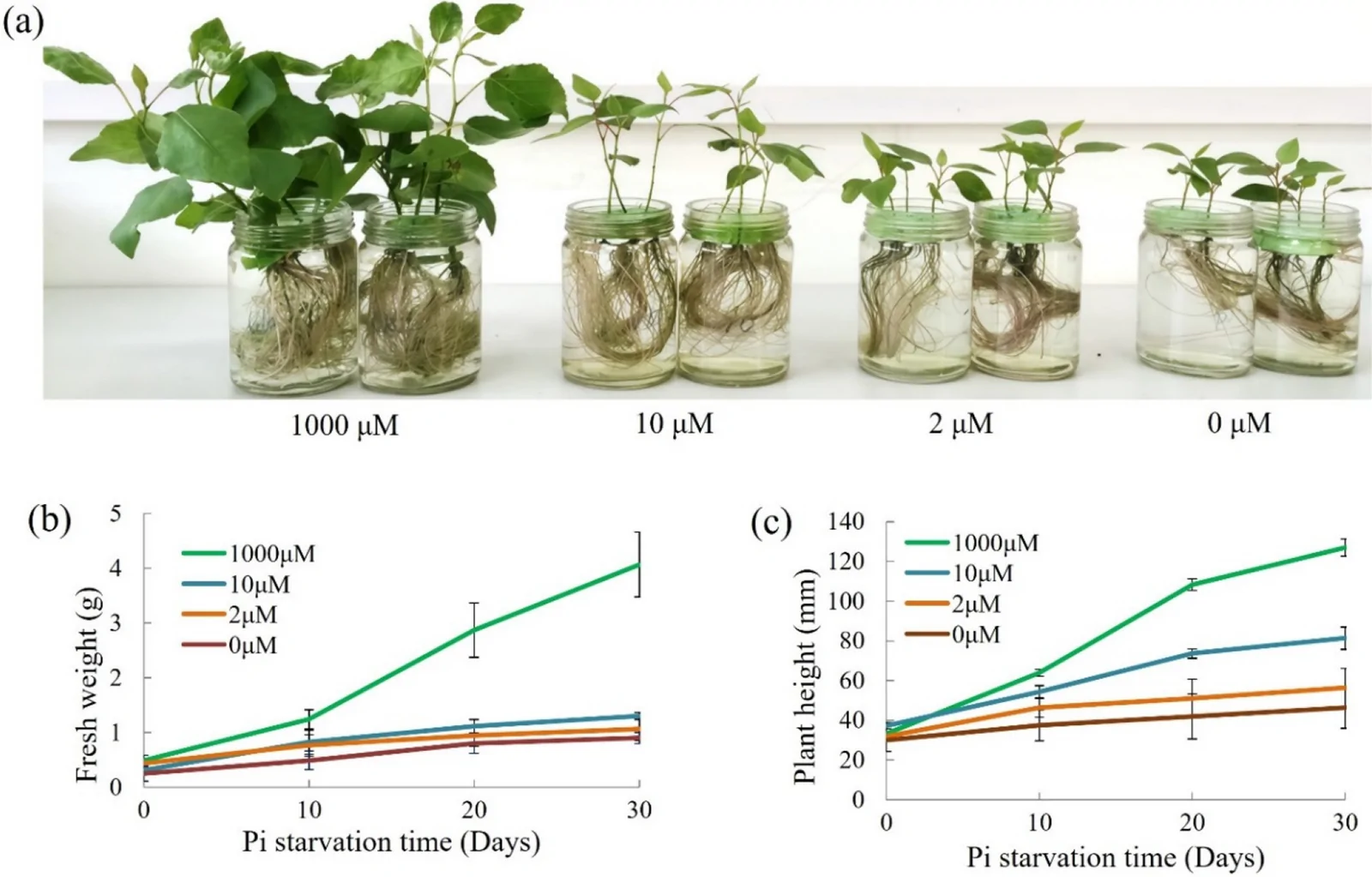
Pi starvation severely restricts poplar growth. **a** The phenotype of 30-day-old poplar plants grown in hydroponic medium with different concentrations of Pi. **b** The fresh weight and (**c**) height of plants at different times. Error bars represent SE (*n* = 3). Three independent experiments were conducted and showed a similar trend, and the representative results are shown

We first assessed the impact of Pi deficiency on tree growth using poplar (*Populus deltoides* x *P. euramericana* NL895). To this end, we grew developmentally synchronized seedlings in hydroponic medium with different concentrations of Pi. Previous studies have shown that the commonly used normal Pi concentrations in experiments are around 1 mM (641 μM to 1.25 mM), while the available Pi ranges from 0.6 to 11 μM in soil solution (Lambers and Plaxton 14. Therefore, a series of Pi concentrations (0, 2, 10 and 1000 μM) was set for Nanlin 895 to investigate its growth response to varying Pi levels. Plants grown under sufficient Pi conditions (1000 µM) exhibited vigorous shoot growth and continuously accumulated biomass (Fig. 1a, b and c). By contrast, plants grown at low Pi (< 10 µM) displayed strongly inhibited shoot growth, as demonstrated with smaller leaves, reduced height and biomass accumulation. Furthermore, their growth was almost halted when Pi starvation extended for more than twenty days (Fig. 1b and c). Thus, Pi deficiency could dramatically suppress poplar growth and decrease productivity.

### PdePHR1 is a MYB-CC protein encoding gene

**Fig. 2:**
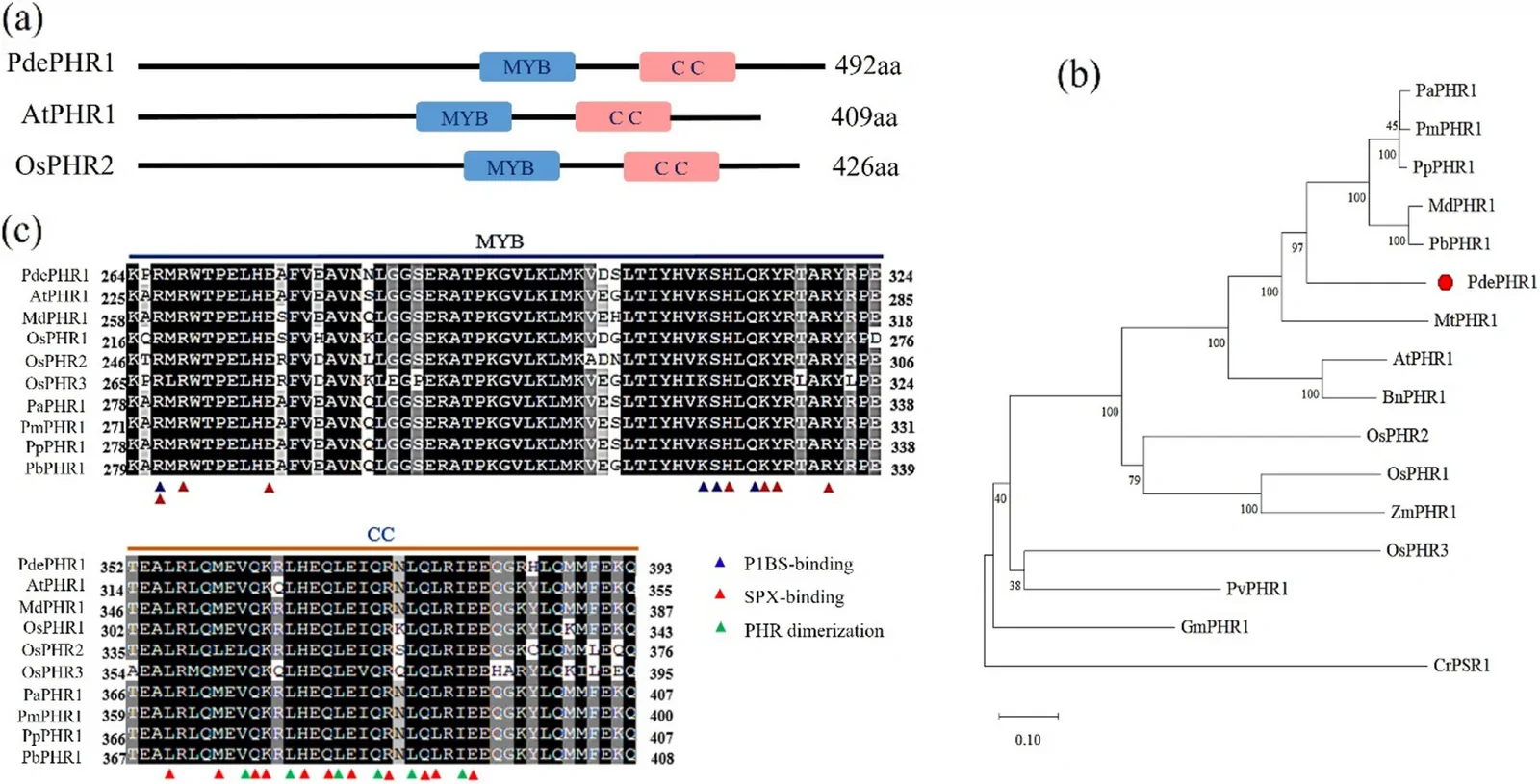
PdePHR1 harbors structure features conserved in PHR1 homologs. **a** schematic structures of PdePHR1 along with AtPHR1 and OsPHR2. The conserved MYB and CC domains are indicated. The numbers depict the length of each protein. **b** Phylogenetic tree of PHR family members generated by MEGA 11 software. **c** Sequence alignment of the MYB (top) and CC (bottom) conserved domains among members of the plant PHR family. Identical amino acids are shaded in black. Triangles indicate the critical residues medicating P1BS-binding (blue), SPX-binding (red), and PHR dimerization (green) [12]. Species code are as follows: *A. thaliana* (At), *Prunus avium* (Pa), *Prunus mume* (Pm), *Prunus persica* (Pp), *Pyrus x bretschneideri* (Pb), *Malus domestica* (Md), *Medicago truncatula* (Mt), *Brassica napus* (Bn), *Oryza sativa* (Os), *Zea mays* (Zm), *Phaseolus vulgaris* (Pv), *Glycine max* (Gm), and *Chlamydomonas reinhardtii* (Cr)

To identify poplar *PHR* gene, we searched the *Populus trichocarpa* genome using *AtPHR1* sequence and found the best hit *Potri.002G257800*. Based on this reference gene sequence, a candidate *PdePHR1* gene was cloned from *P. deltoides* x *P. euramericana* NL895 (Figure S1). Sequence analysis showed that the putative PdePHR1 encompassing 492 amino acid contains a long N-terminal region, a MYB-DNA binding domain and a CC motif (Fig. 2a). A phylogram of homolog proteins from different plant species and counterpart PSR1 in the unicellular algae *Chlamydomonas reinhardtii* was constructed (Fig. 2b), indicating that PdePHR1 is related to CrPSR1 and more closely related to AtPHR1 and OsPHR2 from multicellular vascular plants. Notably, PdePHR1 belongs to the subgroup composing of homologs from woody plants. Furthermore, sequence alignment of the MYB and CC domains showed that PdePHR1 shares critical amino acid residues known to mediate P1BS-binding, SPX-binding and PHR dimerization (Fig. 2c). These structure features provide basis for PdePHR1 to active the expression of target genes by recognizing P1BS *cis*-element as a dimer and be regulated by SPX proteins. Together, these results suggested that PdePHR1 is likely the candidate of the key transcription factor governing PSR in poplar.

### PdePHR1 contains a transcriptional activation domain and is constitutively expressed

**Fig. 3:**
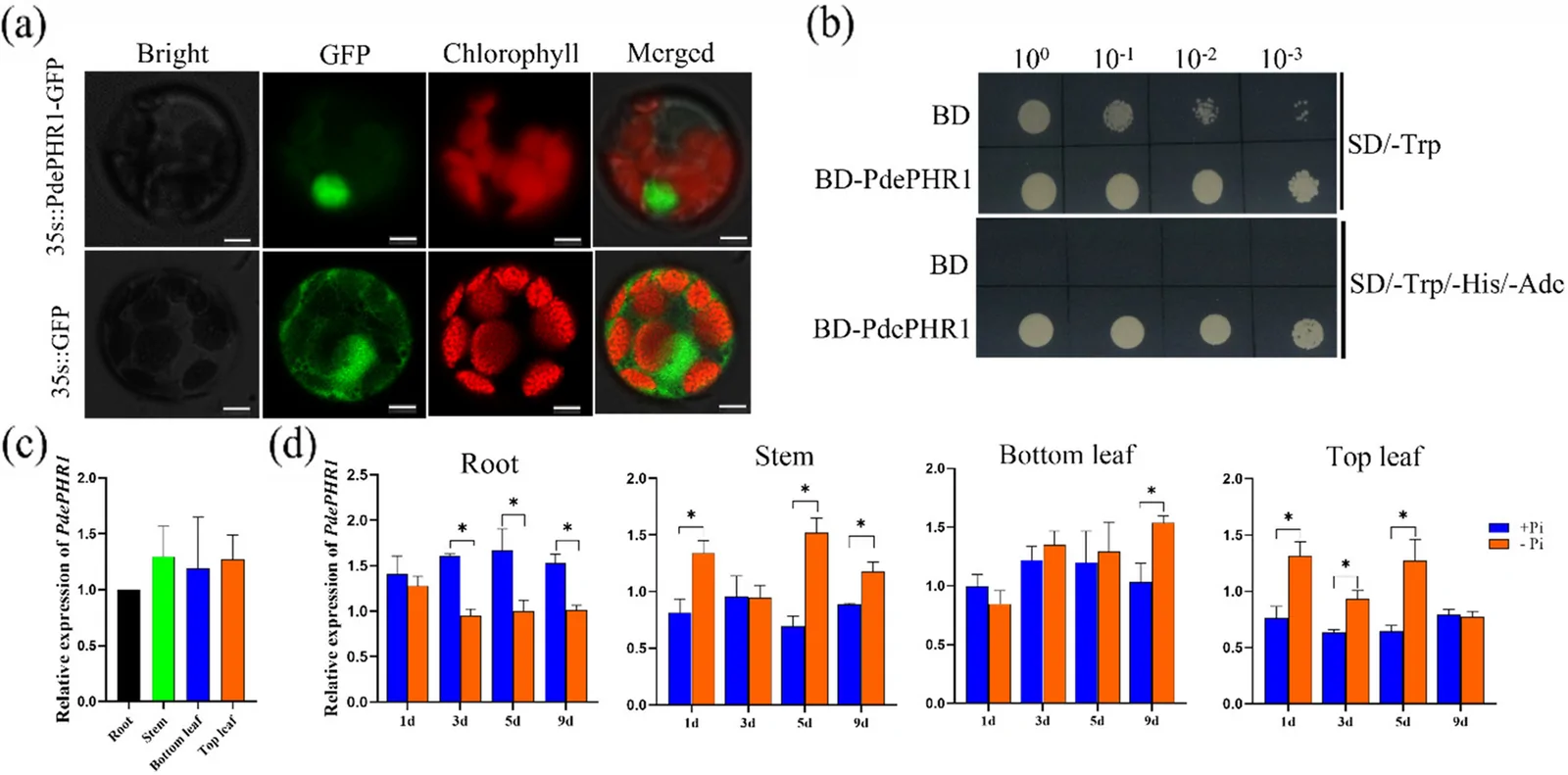
PdePHR1 contains a transcriptional activation domain and is constitutively expressed. **a** The subcellular localization of PdePHR1 protein. **b** Transactivation activity assay. The full-length *PdePHR1* fragment was fused to the GAL4 DNA binding domain to construct the *pBD-PdePHR1* vector and transformed to yeast. BD refers to the negative control *pGBKT7* vector. The transformed strains were grown on the medium of SD/− Trp and selected on the medium of SD/-Trp-His-Ade. **c** Tissue-specific expression of *PdePHR1* in Pi sufficient conditions. Data were normalized against root with *EIF4B* as an internal control. **d** Time-series transcriptional profiling of *PdePHR1* across different tissues and in response to Pi-deficient conditions. Data were normalized against 0 d with *EIF4B* as an internal control. Error bars represent SE. The experiments were repeated three times. *p < 0.05, ** p < 0.01, *** *p* < 0.001, one-way ANOVA(c) or two-way ANOVA (d) was used for statistical analysis. Scale bars, 5 µm

Next, we examined the subcellular localization of PdePHR1. The full-length coding sequence (CDS) of *PdePHR1* was fused to the green florescence protein (*GFP*). For protoplasts transformed with the *35S::GFP* vector, GFP protein was present throughout the cell. In contrast, the fluorescence signal of PdePHR1-GFP was mainly found in the nucleus, suggesting that PdePHR1 is a nuclear protein (Fig. 3a). We then asked whether PdePHR1 protein possesses transcriptional activity. To this end, we performed an autonomous gene activation assay in the yeast system. The full-length *PdePHR1* fragment was fused to the GAL4 DNA binding domain to construct the *pBD-PdePHR1* vector and transformed to yeast. As shown in Fig. 3b, both yeast strains containing either empty *pBD* or *pBD-PdePHR1* grew well in SD/-Trp, indicating a successful transformation of these vectors. However, only the presence of PdePHR1 could support yeast growth on SD/-Trp/-His/-Ade triple nutrition-deficient selective media. The results demonstrated that PdePHR1 contains a transcriptional activation domain and might functions as a transcriptional activator.

We further investigated whether Pi stress affects *PdePHR1* transcription by quantifying its transcript levels. Samples were collected from different poplar organs at multiple time points during Pi starvation. As shown in Fig. 3c, *PdePHR1* mRNA was detected across roots, stems, bottom and top leaves. In general, aerial tissues showed relatively higher abundance of *PdePHR1* transcripts in normal conditions. When subjected to Pi-deficient conditions, the transcription of *PdePHR1* in poplar plants was weakly responsive (Fig. 3d). These expression patterns are in line with the characters of *AtPHR1* and *OsPHR2* [26, 38]. Collectively, these results indicated that PdePHR1 is a nuclear protein and contains a transcriptional activation domain. Concomitantly, *PdePHR1* is only slightly responsive to Pi starvation in bottom leaf, implying a post-transcriptional regulatory mechanism of PdePHR1 activity.

### PdePHR1 overexpression promotes poplar adventitious root growth

**Fig. 4:**
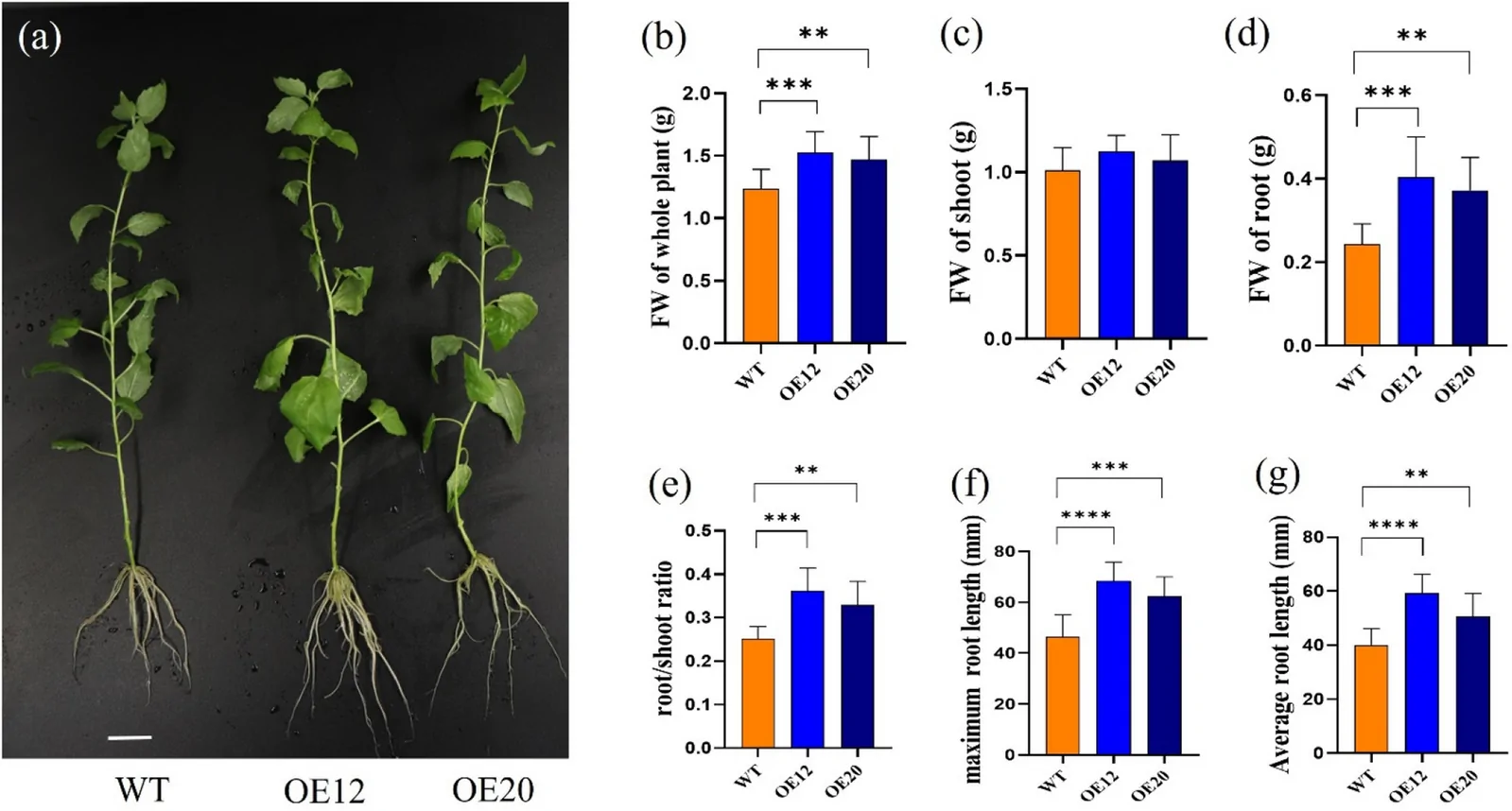
*PdePHR1 *overexpression promotes poplar adventitious root growth in Pi-sufficient conditions. **a** The phenotype of 8-week-old wild type (WT), OE12, and OE20 plants grown in Pi replete medium. **b** - **d** Fresh weight of whole plants (**b**), shoots (**c**) and roots (**d**). **e** The root-to-shoot ratio of the fresh weight. **f** and **g** The maximum length (**f**) and average length (**g**) of adventitious roots. Data are shown as means ± SE, (*n* = 12 for WT, *n* = 15 for OE12, and *n* = 13 OE20). Three independent experiments were conducted and showed a similar trend, and the representative results are presented. Data significantly different from the corresponding controls are indicated (OE versus the WT, **p < 0.01, *** *p* < 0.001, and ****p < 0.0001). The *p* values were determined using one-way ANOVA. Scale bar in (**a**) is 2 cm. FW, fresh weight

Previous results showed that loss of function mutation of AtPHR1 impairs several Pi starvation responses, including alteration of root to shoot growth ratio (Bustos R. et al., 2010). Owing to the low genetic transformation efficiency of Nanlin 895, we generated transgenic plants expressing *35S::PdePHR1-GFP* in *P. davidiana* x *P.bolleana* to explore its role on tree performance. Two independent transgenic lines showed strong expression of *PdePHR1* (Figure S2), designated as *PdePHR1-OE12* and *PdePHR1-OE20*, were used to further experiments. When grew in the tissue culture flask supplied with replete Pi, OE plants showed a significant increase in fresh weight compared with the wild type (WT) (Fig. 4a, 4b). Notably, this was associated with only slight increase in shoots (Fig. 4c). In contrast, the fresh weight of OE roots was remarkably elevated by 52%−59% (Fig. 4d). Consequently, this led to the root-to-shoot ratio greatly shifted from 0.25 for the WT plants up to 0.36 for the OE plants (Fig. 4e), demonstrating a substantially larger amount of biomass accumulation towards underground tissues. In accordance with this, OE plants exhibited considerably longer (34%−47% increase) adventitious roots than those of WT (Fig. 4f, 4 g). Thus, overexpressing *PdePHR1* strongly drives root elongation in Pi replete conditions, suggesting that PdePHR1 might regulate root growth and development in poplar.

### Overexpressing *PdePHR1* increases P content and accumulation in poplar shoots

**Fig. 5:**
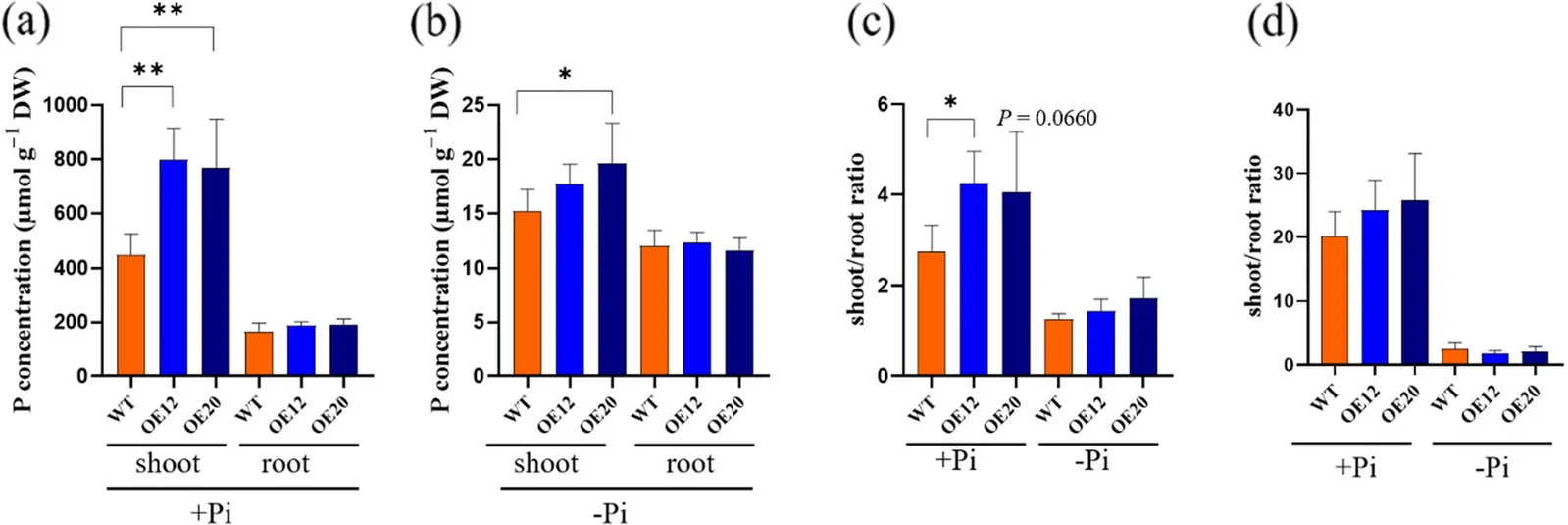
*PdePHR1* overexpressing lines accumulate more P in their shoots under Pi-sufficient conditions. **a** and (**b**) The total P concentrations of the shoot and root of WT and OE poplar plants grown under Pi sufficiency (**a**) and deficiency (**b**) for 6 weeks. **c** The shoot-to-root ratio of total P concentrations of WT and OE plants, respectively. **d** The shoot-to-root ratio of total P content of WT and OE plants, respectively. Data are shown as means ± SE, pooled from three independent experiments with a similar trend (n = 30, pooled from three independent experiments). Data significantly different from the corresponding controls are indicated (OE versus the WT, *p < 0.05, **p < 0.01). The *p* values were determined using two-way ANOVA. DW, dry weight

To determine whether the promotion of poplar growth by overexpressing *PdePHR1* was associated with elevated Pi acquisition, we measured their total P concentrations of plants grown under Pi-sufficient and deficient conditions, respectively. Under Pi sufficiency, P concentrations in Pi-sufficient WT shoots and roots were 449.8 and 166.1 µmol g^–1^ dry weight (DW), respectively (Fig. 5a). Thus, the shoot/root Pi concentration ratio was 2.7 in WT plants, reflecting the normal Pi distribution pattern in poplar (Fig. 5c). For the *PdePHR1* OE lines, P concentrations ranged from 769.5 to 800.0 µmol g^–1^ DW in shoots, and 187.8 to 193.0 µmol g^–1^ DW in roots (Fig. 5a). These data highlight an increase of at least 71% of total P in shoots, and marginally increase in roots by PdePHR1. As a result, the shoot/root P concentration ratio become approximate 4.0 (Fig. 5c), indicating a pronounced shift of P accumulation towards shoot from root. Under Pi-deplete condition, P concentrations of WT shoots and roots dramatically reduced to15.2 and 12.0 µmol g^–1^ DW, respectively (Fig. 5b). Similarly, *PdePHR1* OE lines showed a comparable concentration in roots (11.6 to 12.4 µmol g^–1^ DW) and a slight increase in shoots (17.8 to 19.7 µmol g^–1^ DW) (Fig. 5b). Accordingly, the shoot/root P concentration ratio dropped to 1.3 in WT and 1.4 ~ 1.7 in *PdePHR1* OE plants (Fig. 5c), implying a significant proportion of P is reallocated in roots under Pi starvation stress. The ratio of P content between shoot and root was higher in OE plants in Pi sufficient conditions (Fig. 5d). Taken together, PdePHR1 sufficiently stimulates P accumulation in shoots under Pi replete conditions. Whereas in Pi deprived conditions, both *PdePHR1* OE and WT plants tend to prioritize root demand for P.

### *PdePHR1* overexpression improves plant growth under Pi deficiency in poplar

**Fig. 6:**
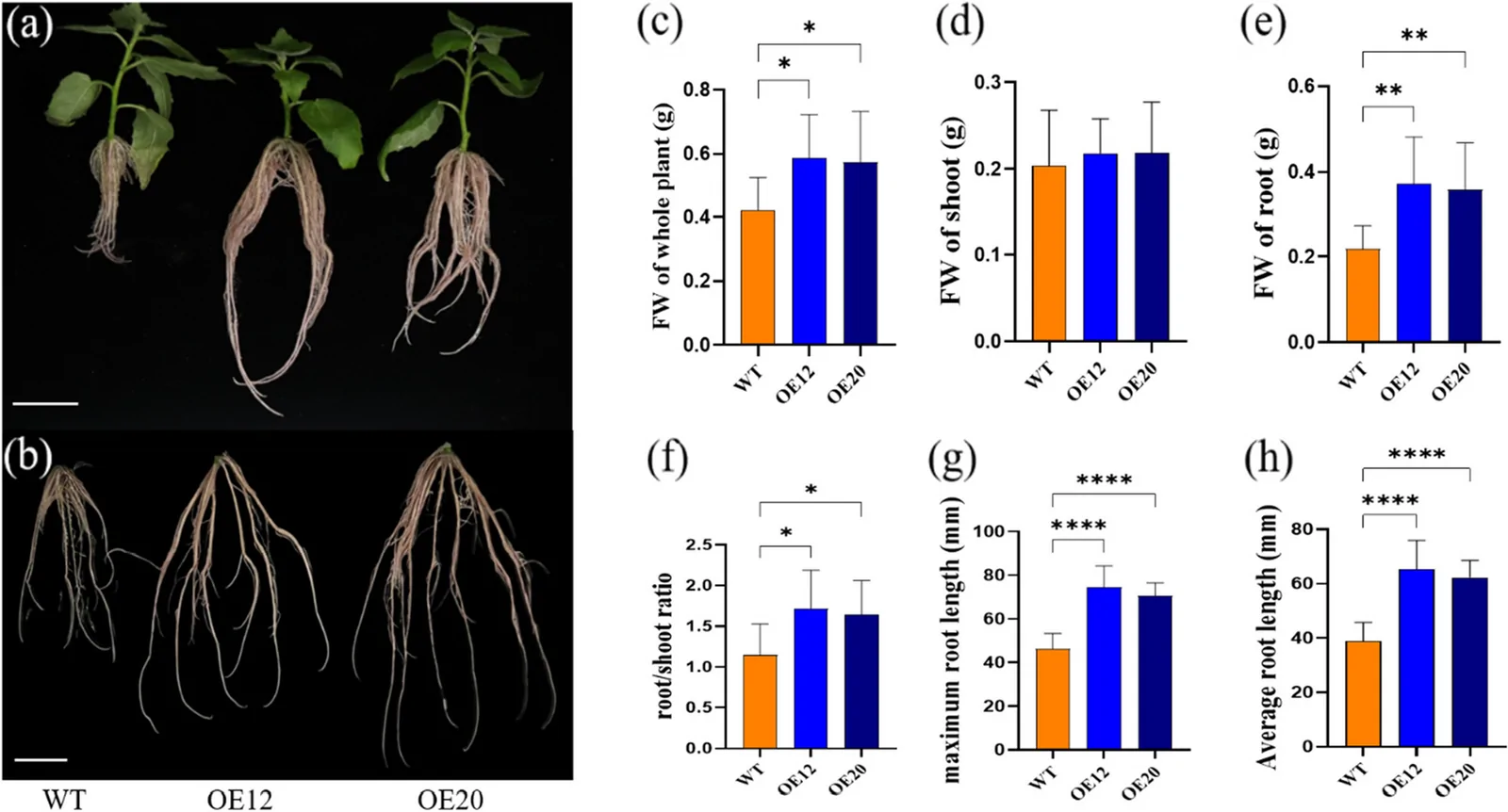
*PdePHR1* overexpression improves plant growth under Pi deficiency in poplar. **a** The phenotype of WT, OE12, and OE20 plants subjected to Pi starvation. The 25-day-old healthy seedlings were transferred to -Pi medium and grew for 30 days. **b** root phenotype. **c**-**e** Fresh weight of whole plant (**c**), shoot (**d**) and root (**e**). **f** The root-to-shoot ratio of fresh weight. **g** and **h** The maximum length (**g**) and average length (**h**) of adventitious roots. Data are shown as means ± SE, pooled from three independent experiments with a similar trend (*n* = 10 for WT and *n* = 12 for OE12 and OE20). Data significantly different from the corresponding controls are indicated (OE versus the WT, *p < 0.05, ** *p* < 0.01, ***p < 0.001, and ****p < 0.0001). The *p* values were determined using one-way ANOVA. Scale bars in (**a**) and (**b**) are 2 cm. FW, fresh weight

Because OE and WT plants exhibited similar low P levels under Pi deprivation, we tested whether *PdePHR1* overexpression could confer poplar better performance during Pi starvation. In flask culture, wild type and *PdePHR1* OE plants presented a clear symptom of Pi starvation with retardation of shoot growth (Fig. 6a). Unsurprisingly, the fresh weights between two genotypes were comparable in shoots (Fig. 6d). Nevertheless, *PdePHR1* OE plants developed dramatically stronger root system in comparison with WT, as evidenced by a 98%−100% greater fresh weight than WT plants (Fig. 6b and e), accounting for the increase in whole plant weight (Fig. 6c). Accordingly, this led to a 1.6 fold higher root-to-shoot ratio of *PdePHR1* OE plants (approximately 1.8) in comparison with WT (about 1.1), manifesting a key adaptive trait for Pi starvation tolerance (Fig. 6f). Consistent with the increase of root mass, *PdePHR1* OE plants displayed substantially longer adventitious roots (Fig. 6a, b, g-h). The increase in the average length of adventitious roots by overexpressing *PdePHR1* was 57%−82% (Fig. 6h). Taken together, these data indicate that PdePHR1 could promote root growth independent of Pi availability and might confer poplar an enhanced Pi deficiency tolerance.

### Overexpressing *PdePHR1* augments poplar productivity under long-term Pi deficiency in green house

**Fig. 7:**
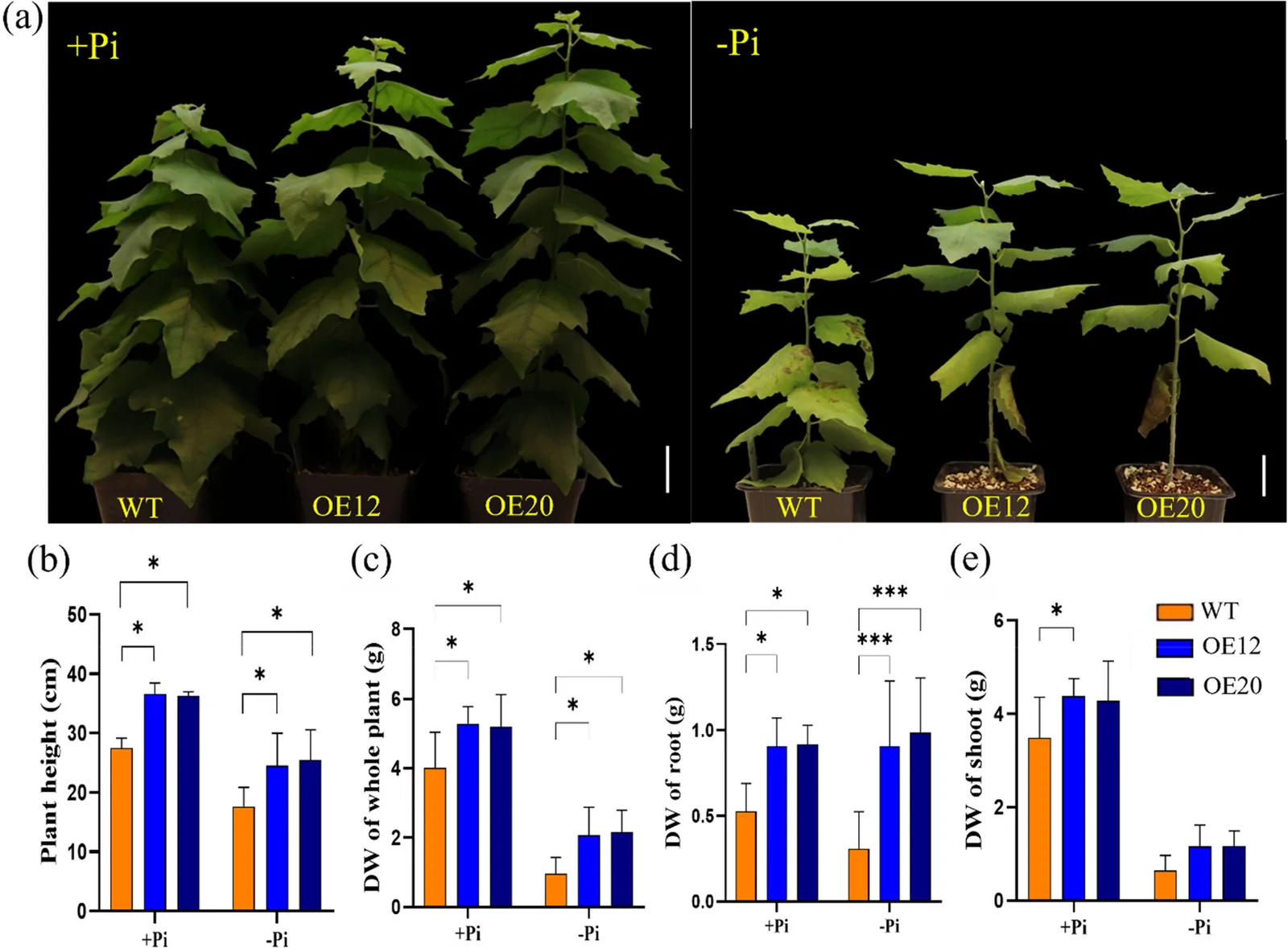
Overexpression of *PdePHR1* confers poplar improved performance under Pi sufficiency and a long-term exposure to Pi starvation in green house. **a** The phenotype of WT, OE12, and OE20 plants grown for two months in green house under + Pi (left) and -Pi (right) conditions, respectively. **b** Plant height. **c** - **e** Dry weight of whole plant (**c**), root (**d**), and shoot (**e**). Data are shown as means ± SD, pooled from three independent experiments with a similar trend (*n* = 6 for each genotype under Pi sufficiency conditions; *n* = 10, 8 and 8 for WT, OE12, and OE20, respectively, under Pi deficiency. Data significantly different from the corresponding controls are indicated (OE versus the WT, *p < 0.05, ***p < 0.001). The *p* values were determined using two-way ANOVA. Scale bars in (**a**) is 4 cm. DW, dry weight

The flask cannot support fast growing poplar tree to grow fully due to size limitation. To assess the performance of OE lines in greenhouse for a longer period, we next grew both WT and OE cuttings in greenhouse under either sufficient or deficient Pi conditions for two months. Surprisingly, *PdePHR1* OE plants showed a significant increase in height compared with WT under Pi sufficient conditions (Fig. 7a, 7b). This was not evident for the seedlings cultured in the space-limited flasks (Fig. 4a). Consistent with previous observations, the *PdePHR1* OE poplar plants accumulated more biomass in relative to WT plants (Fig. 7c). In particular, the dry weight of OE roots was about 1.6 times that of WT under normal conditions (Fig. 7d). When grew under Pi starvation, the plant productivities of all genotypes were significantly reduced (Fig. 7a-c). Meanwhile, OE exhibited taller than WT plants (Fig. 7a-b). On the contrary, the root biomass was boosted in both *PdePHR1* OE and WT plants, with the former accumulated more than 54% than the latter (Fig. 7d), demonstrating a great improvement in root growth driven by PdePHR. On the other hand, the biomass of *PdePHR1* OE shoots showed a marginal increase (Fig. 7e). These observations confirm that PdePHR1 strongly stimulates root system growth and development and increases biomass accumulation in woody tissues. Moreover, overexpression of *PdePHR* is sufficient to confers poplar greater productivity in optimal conditions and better adaptation to Pi scarce environment.

### PdePHR1 is involved in upregulating the expression of PSR genes in Pi-sufficient conditions

**Fig. 8:**
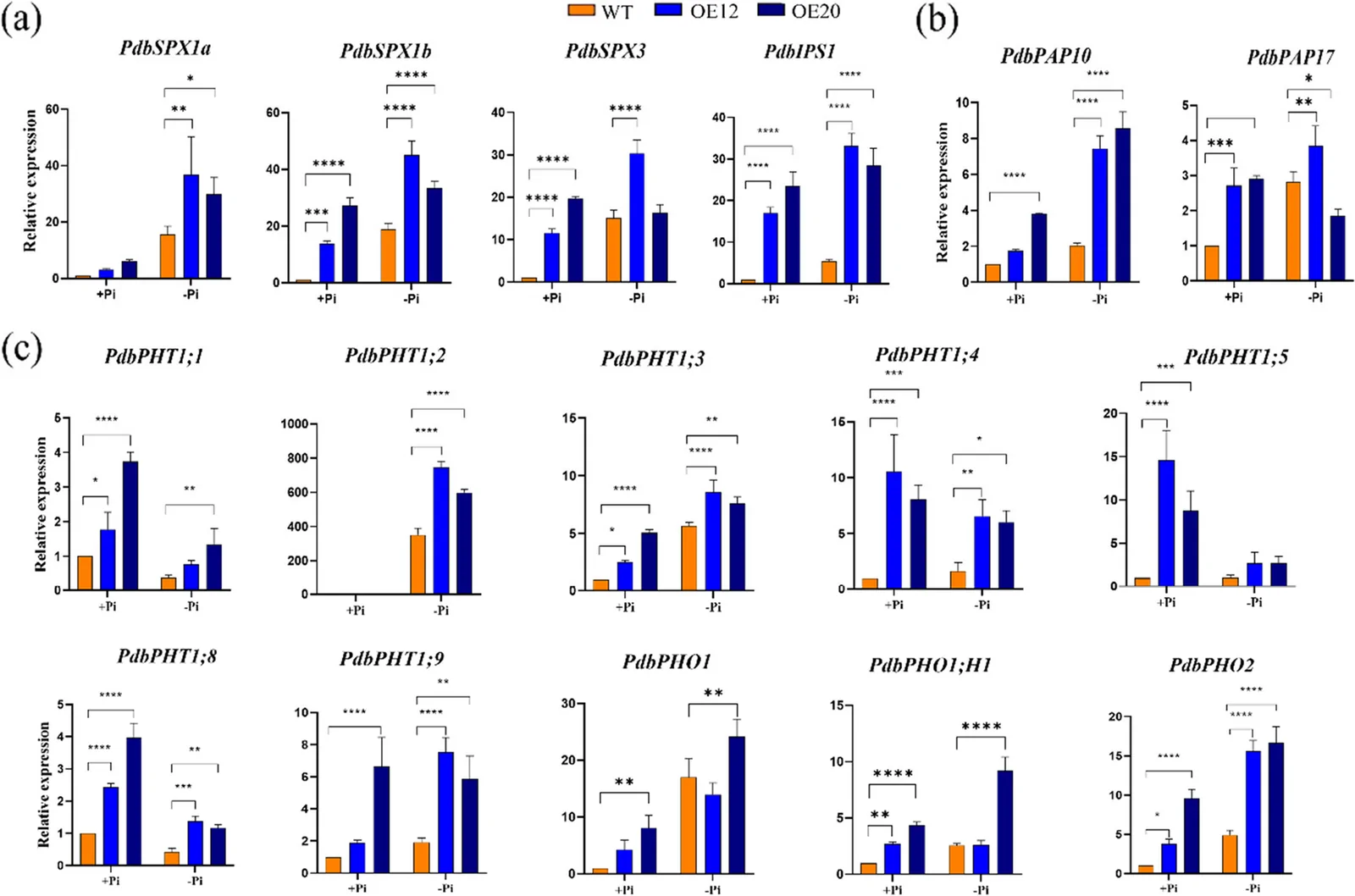
*PdePHR1 *activates transcription of PSR genes in Pi-sufficient conditions. **a** - **c** The relative transcription levels of key components involving in Pi starvation signaling pathway (**a**), remobilization (**b**) and transport (**c**). The seedlings grown on + Pi medium for 10 days, and then were transferred to -Pi medium or + Pi medium, respectively, for 3 days. Data were normalized against EIF4B. The values are means ± SD. Three independent experiments were conducted and showed a similar trend, and the representative results are presented. Data significantly different from the corresponding controls are indicated (OE versus the WT, *p < 0.05, **p < 0.01, ***p < 0.001). The *p* values were determined using two-way ANOVA. *SPX domain-containing proteins* (*SPX*s); *purple acid phosphatases* (*PAP*s); *PHOSPHATE TRANSPORTER1* (*PHT1*); *PHOSPHATE 1* (*PHO1*); *PHOSPHATE 2* (*PHO2*)

Since PdePHR1 possessed transcriptional activation activity and promoted P accumulation in Pi-sufficient conditions, we hypothesized that PdePHR1 could trigger transcription of PSR genes in poplar. We used OE lines to measure the transcript abundances of different types of PSR genes involved in Pi signaling, remobilization and transport. The developmentally synchronized WT and OE poplar seedlings were transferred onto Pi-sufficient or deficient media for 3 days. *SPX1*, *SPX3* and *IPS1* are key components relaying Pi starvation signal and their transcriptions are induced by AtPHR1. We found their poplar homologs by searching the *P. trichocarpa* genome (Figure S3). We then examined the expression profiles of these poplar homologs including *SPX1a, SPX1b, SPX3* and *IPS1*. The transcriptional levels of these four genes were all induced by Pi deficiency in the WT plants, suggesting they are indeed PSR genes in poplar. More importantly, the expression levels of *SPX1b*, *SPX3* and *IPS1* in *PdePHR1* OE lines were remarkably induced compared with that of WT in Pi-sufficient conditions (Fig. 8a). In addition, their transcription levels were further elevated compared to the WT in Pi-deficient conditions. (Fig. 8a), suggesting that PdePHR1 activates the expression of *SPX1*s, *SPX3*, and *IPS1* irrespective of Pi availability in the environment. For genes involved in Pi remobilization, we focused on the purple acid phosphatases genes *PAP10* and *PAP17.* Similarly, AtPAP10 and PAP17 were used to find their poplar homologs (Figure S3) [15]. The expression of two *PAP* homolog genes were both induced upon Pi deficiency and upregulated by overexpressing *PdePHR1* under Pi sufficiency as well (Fig. 8b). Among seven *PHT1* family genes, the expression of *PHT1;2, PHT1;3, PHT1;4,* and *PHT1;9* was induced in response to Pi starvation. Whereas the transcription of *PHT1;4* and *PHT1;5* did not respond to Pi starvation in the WT root, implying that they might play role in normal conditions (Fig. 8c). Strikingly, despite replete Pi supply, transcription levels of all seven *PHT1* members were elevated in *PdePHR1* OE plants compared with that of WT (Fig. 8c). Notably, their expression levels were also higher in OE than WT roots under Pi deprived conditions. These results clearly indicate that PdePHR1 positively regulate *PHT1* genes, which is in line with the increased P accumulation in OE plants. PHO1 and its close homolog PHO1;H1mediate Pi translocation from root to shoot. In general, the transcript abundance of *PHO1* and *PHO1;H1* is higher in OE roots than WT on Pi-sufficient medium (Fig. 8c), consisting with higher P concentration in OE shoots. Notably, the activity of PHO1 and PHT1 is controlled at the transcriptional or at the translational level by PHO2. PHO2 mediates the degradation of PHT1s and PHO1 [11, 17]. *PHO2* expression level in *PdePHR1* OE lines was more abundant compared to the WT on both + Pi and -Pi medium (Fig. 8c), implying that a regulatory loop involving transcriptional control of *PHO2* by the shoot P concentration. Higher shoot P level might increase *PHO2* expression, which would subsequently enhance PHO1 degradation. Thereby, OE plants could prevent Pi over-accumulation in the shoot and secure Pi retention in the root. Thus, this seemingly contradictory expression pattern might suggest a PdePHR1-mediated fine regulation of Pi distribution between roots and shoots through manipulating *PHO2*. Taken together, our results reveals that PdePHR1 drives the activation of key PSR genes in both Pi regimes, contributing to the enhanced P accumulation, fine regulation of P homeostasis and better performance of OE plants.

## Discussion

As an indispensable component of genetic molecule, membrane structure, metabolic fuel and reactant, Pi inadequacy hinders plant growth [21, 28]. Fast-growing trees are of high demand for P to build up massive biomass. Consistently, Pi limitation could lead to significantly stunted growth and reduced biomass accumulation in poplar tree (Fig. 1). Particularly, Pi deficiency greatly suppressed poplar stem growth, while substantially stimulated the growth of adventitious roots (Figs. 6, 7). Here we identified that PdePHR1 contains a transcriptional activation domain and might function as a master transcriptional activator to modulate P accumulation and distribution, and root growth.

PdePHR1 is the central regulator of Pi starvation responses in poplar. This notion is supported by its structure features, molecular characters and physiological functions. Firstly, PdePHR1 shares a characteristic MYB-CC domain with AtPHR1 and OsPHR2 [29, 38] (Fig. 2a, b). More importantly, all the previously identified critical amino acid residues that involved in PHR dimerization, target DNA binding and SPX interaction are perfectly conserved in PdePHR1 as well (Fig. 2c), providing the structural foundation for its functionality in Pi starvation response [12, 13]. Secondly, PdePHR1 localizes in the nucleus and possesses a transcriptional activation domain (Fig. 3a, b). Indeed, PdePHR1 could transcriptionally activate different types of PSR genes including signaling, transporters and catabolism (Fig. 8). Additionally, PdePHR1 is weakly responsive to external Pi status (Fig. 3c), which is consistent with the expression pattern of *AtPHR1* and *OsPHR2* [26, 38], implying a similar posttranscriptional regulation mechanism in response to Pi deficiency. Meanwhile, the presence of putative SPX homologs also points to conserved mechanisms of intracellular Pi sensing via posttranslational SPX-PHR signaling pathway in poplar. Thirdly, PdePHR1 overexpression poplar plants manifest increased root-to-shoot ratio, a conventional PSR in plants, despite abundant Pi supply (Fig. 4e). Furthermore, PdePHR1 overexpressing plants accumulate substantially greater P in shoots than that of WT plants under Pi replete conditions (Fig. 5), suggesting an enhanced Pi absorption and subsequent root-to-shoot translocation. These observations are in agreement with previous reports in Arabidopsis and rice [22, 38]. Moreover, our findings reveal that overexpressing PdePHR1 alone is sufficient to drive the expression of PSR genes and increase P acquisition and reallocation as well, suggesting that PdePHR1 could contribute to the tree adaption to the environmental Pi limitation and sustain their productivity.

PdePHR1 might involve in facilitating stem growth. Notably, our data and previous studies show that high expression of PHR is invariably associated with high P concentrations in shoots of both herbaceous plants and poplar grown under sufficient Pi supply [22]. Interestingly, *AtPHR1* and *OsPHR2* overexpression lines display Pi toxicity phenotypes, such as leaf senescence and lower height, at the replete Pi conditions [22, 36, 38]. Conversely, *PdePHR1* overexpressing poplar plants exhibit vigorous shoot growth manifesting increased height (Fig. 7a, b). This difference in shoot performance might be attributed to indefinite growth of trees, by which shoots utilized the excess Pi. Intriguingly, *OsPHR2*-overexpression rice plants displayed lower height than WT plants under both Pi sufficiency and deficiency [31], demonstrating that additional factor may be involved in shoot inhibition by *OsPHR2* overexpression alongside Pi accumulation. Further experiments revealed that PHR2 induces the expression of *MYB110*, a negatively regulator of rice height, thereby inhibits shoot growth irrespective of Pi supplies [31]. Given the fact that *PdePHR1* overexpression increases poplar shoot height, the PHR2-MYB110 module cannot explain the contrast shoot phenotypes. Thus, this module is unlikely present in poplar, or a different PHR-mediated downstream regulatory mechanism might operate independent of PHR function in governing Pi accumulation.

The function of PdePHR1 in promoting root growth is independent of the regulatory role of Pi homeostasis in poplar. Adjusting root growth is a common adaptive response of plants to survive in different ecological niches, including low Pi environment [25]. PHRs function in shaping plant root architecture. OsPHR2 has been reported to regulate root architectural alteration in response to Pi levels [38]. In Pi-deficient conditions, the primary and adventitious root lengths of *OsPHR2* overexpression rice plants are longer than those of WT plants. By contrast, overexpressing *OsPHR2* leads to decreased biomass and slightly shorter primary root under sufficient Pi supply. Different with rice, overexpression of *PdePHR1* profoundly stimulates poplar adventitious root elongation and consequential biomass accumulation under both replete (Figs. 4, 7) and depleted Pi conditions (Figs. 6, 7). Our results clearly suggest that PdePHR1 acts as a positive regulator of adventitious root growth irrespective of external Pi status. How PdePHR1 integrate with the complex root system growth and development process remains to be explored.

The genetic evidence from *PdePHR1* OE poplar shows that *PdePHR1* alone is sufficient to boost root system to a great extent. In addition, *PdePHR1* overexpression poplar plants favor root growth, as evidenced by greater root-to-shoot ratio (Figs. 4e, 5f), implying a much slower carbon decay compared to leaves. Taken together, trees with enhanced *PdePHR1* expression develop massive deeper roots might have better fitness in fluctuating environments, as well as increased C sequestration in the soil. A deeper root system might help to withstand the force of rain and wind as well.

## Conclusions

PdePHR1 serves as a master regulator of Pi homeostasis and root growth in poplar, whose overexpression boosts woody biomass accumulation regardless of environmental Pi availability. These findings facilitate the understanding of the mechanisms of phosphate homeostasis in forest trees and provide a novel target for breeding and transgenic approaches to generate woody plants with improved adapation to suboptimal environments and subsequent carbon sequestration in the soil.

## Supplementary Information

Supplementary Material 1.

Supplementary Material 2.

Supplementary Material 3.

Supplementary Material 4.

## Data Availability

Data sharing is not applicable to this article as no datasets were generated or analysed during the current study.
