# Supplementary material for: The transcription factor PdePHR1 increases phosphorus accumulation in poplar shoot and promotes root growth
Source: BMC Plant Biol. 2026 Jul 24;26:1805. doi: 10.1186/s12870-026-09584-6 (PMC13685269; doi:10.1186/s12870-026-09584-6)
Supplement: Supplementary file 1 [file 12870_2026_9584_MOESM3_ESM.docx]

**Figure S3. Sequence alignments of PSR genes used in Figure 8**

PHO1;H11

PHO1

PAP17

IPS1

PHT1;1

PHT1;2

PHT1;3

PHT1;4

PHT1;5

PHT1;8

PHT1;9

PAP10

PHO2

SPX1

SPX3

Supplemental Figure S3. q-RT PCR involves homologous alignment results between genes and related genes in Arabidopsis thaliana
